# Facile synthesis of surface-functionalized magnetic nanocomposites for effectively selective adsorption of cationic dyes

**DOI:** 10.1186/s11671-018-2476-7

**Published:** 2018-04-12

**Authors:** Yani Hua, Juan Xiao, Qinqin Zhang, Chang Cui, Chuan Wang

**Affiliations:** 1 Key Laboratory of Reservoir Aquatic Environment, Chongqing Institute of Green and Intelligent Technology, Chinese Academy of Sciences, Fangzheng Avenue, number 266, Beibei District, Chongqing, 400714 China; 2 University of Chinese Academy of Sciences, Beijing, 100049 China; 3 Guangdong Environmental Monitoring Center, Guangzhou, 510308 China; 4 Yangtze Normal University, Chongqing, 400714 China; 5 School of Environmental Science and Engineering, Guangzhou University, Guangzhou, 510006 China

**Keywords:** Adsorption, Polycatechol, Fe_3_O_4_, Magnetic adsorbent, Selectivity

## Abstract

A new magnetic nano-adsorbent, polycatechol modified Fe_3_O_4_ magnetic nanoparticles (Fe_3_O_4_/PCC MNPs) were prepared by a facile chemical coprecipitation method using iron salts and catechol solution as precursors. Fe_3_O_4_/PCC MNPs owned negatively charged surface with oxygen-containing groups and showed a strong adsorption capacity and fast adsorption rates for the removal of cationic dyes in water. The adsorption capacity of methylene blue (MB), cationic turquoise blue GB (GB), malachite green (MG), crystal violet (CV) and cationic pink FG (FG) were 60.06 mg g^− 1^, 70.97 mg g^− 1^, 66.84 mg g^− 1^, 66.01 mg g^− 1^ and 50.27 mg g^− 1^, respectively. The adsorption mechanism was proposed by the analyses of the adsorption isotherms and adsorption kinetics of cationic dyes on Fe_3_O_4_/PCC MNPs. Moreover, the cationic dyes adsorbed on the MNPs as a function of contact time, pH value, temperature, coexisting cationic ions and ion strength were also investigated. These results suggested that the Fe_3_O_4_/PCC MNPs is promising to be used as a magnetic adsorbent for selective adsorption of cationic dyes in wastewater treatment.

**Electronic supplementary material:**

The online version of this article (10.1186/s11671-018-2476-7) contains supplementary material, which is available to authorized users.

## Background

Inorganic and organic wastes produced by human activities have resulted in high volumes of contaminated waters which threaten the health of human beings and other living organisms [1]. Water pollution is one of the most serious environmental problems nowadays, which hinders the development of human society [2, 3]. In particular, dye pollutants have attracted wide concerns from the public because of the high visibility and the toxic impact on biological organisms and the ecology [4]. Organic dyes have been extensively used in various branches such as textile, paper, printing, color photography, pharmaceutical industry, leather, cosmetics, plastic and other industries, which have been the major industrial wastewater sources [5]. The quantity of dye wastewater is extremely large, generally, the volume of discharged wastewater from each step of a textile operation is approximately at a high rate of between 40 L/kg and 65 L/kg of the product [6]. In addition, dyes are non-biodegradable substances that remain stable under different conditions due to their synthetic origin and complex aromatic structures [7]. Therefore, it is necessary to select an appropriate method to remove dyes from wastewater before discharging into the environment.

In recent years, a variety of techniques has been used to treat dye wastewater including photocatalytic degradation [8], coagulation [9], electrochemical processes [10], chemical oxidation [11], membrane filtration [12], biological treatment [13] and adsorption [14]. Among these dye wastewater treatment techniques, adsorption has been widely used due to their merits of simplicity, high efficiency and economy [15, 16]. Many adsorbents such as activated carbon, kaolin, montmorillonite clay, waste red mud, fullers earth and fired clay have been reported to decolorize wastewater [17, 18]. Especially, magnetic nanoparticles (MNPs) have attracted considerable attention as adsorbent materials for organic dyes and heavy metals, due to their unique magnetic properties, low cost, biocompatible, easily synthesized, readily recycle, particularly economic and environmental friendly [19]. Several methods have been developed to synthesize magnetic Fe_3_O_4_ nanoparticles, including i) coprecipitation of ferrous and ferric aqueous solution in the presence of a base [20]; ii) thermal decomposition of an iron complex [21]; iii) sonochemical approach [22].

Because of their high surface energies and intrinsic magnetic interactions, easy aggregation of Fe_3_O_4_ MNPs would reduce their surface/volume ratio and dispersion stability in aqueous solution [23]. The stabilizers such as surfactants, supporters, oxides or polymeric compounds have been used to modify Fe_3_O_4_ MNPs to increase their stability and improve their dispersity. Zhang et al. synthesized magnetic Fe_3_O_4_/C core shell nanoparticles and used as absorbents performing good adsorption capacity for dye removal [24]. Wang et al. prepared Fe_3_O_4_ nanoparticles with cetyltrimethylammonium bromide (CTAB) assistant for adsorption removal of congo red (CR) and methylene blue (MB) [25]. Furthermore, the adsorption capacity of bare Fe_3_O_4_ MNPs is not strong enough.

In order to improve the adsorption property, surface functionalization of Fe_3_O_4_ MNPs has been studied. Zhang et al. modified Fe_3_O_4_ MNPs with 3-glycidoxypropyltrimethoxysilane (GPTMS) and glycine (Gly), the magnetic nanocomposites could excellently remove both anionic and cationic dyes in severe environment (highly acidic or strong alkaline) [26]. Moreover, selective adsorption can be greatly improved for the enrichment of pollutants due to introduction of large numbers of active sites. Pourjavadi et al. reported a new functionalized magnetic nanocomposite of poly(methylacrylate) for the efficient removal of anionic dyes from aqueous media [27]. Polycatechol, resulting from the polymerization of catechol catalyzed by Fe(III) [28–30], has been exploited in surface modifications as adhesives and coatings over a wide range of both organic and inorganic materials due to their unique thermal, structural properties, and the ability to form stable complexes with metal oxides [31, 32]. It means that Fe_3_O_4_ MNPs modified with polycatechol will greatly increase the adsorption ability of Fe_3_O_4_ MNPs. However, there is no report about polycatechol modified Fe_3_O_4_ MNPs as an absorbent for dye removal by far.

In this work, polycatechol modified Fe_3_O_4_ MNPs (Fe_3_O_4_/PCC MNPs) were prepared by a facile coprecipitation method and used as adsorbents for dye removal. The absorbent was characterized using magnetic hysteresis loops, thermogravimetric analysis and zeta potential analysis technique. Five kinds of cationic dyes, including methylene blue (MB), cationic turquoise blue GB (GB), malachite green (MG), crystal violet (CV) and cationic pink FG (FG), were chosen as the model compounds to expose the adsorption behavior of Fe_3_O_4_/PCC MNPs. The adsorption kinetics, isotherm analyses and the effect of different experimental conditions on the removal of cationic dyes were also investigated.

## Methods

### Materials

**Fig. 1:**
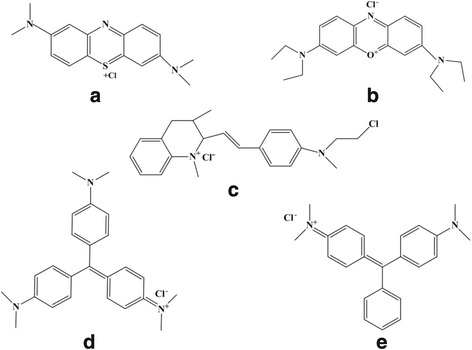
Molecular structures of (**a**) MB (**b**) GB (**c**) MG (**d**) CV (**e**) FG. As shown as Fig. 1, the structure of five kinds of cationic dyes are described

Ferric chloride (FeCl_3_·6H_2_O), ferrous sulfate (FeSO_4_·7H_2_O), ammonium hydroxide (NH_3_·H_2_O, 25%), MB, GB, MG, CV, FG, Orange ΙΙ, Fuchsin, methyl orange (MO) and catechol were obtained from Chuandong Chemical Inc., Chengdu, Sichuan, China. All chemicals were analytical grade and used without further purification and all solutions and suspensions were prepared with deionized water. The structures of five cationic dyes, including MB, GB, MG, CV and FG, were shown in Fig. 1.

### Preparation and characterization of Fe_3_O_4_/PCC MNPs

Fe_3_O_4_/PCC MNPs were prepared by a facile chemical coprecipitation method using iron salts and catechol as precursors [23]. The whole synthesis process was performed at ambient atmosphere. In brief, FeCl_3_·6H_2_O (10 mmol) and FeSO_4_·7H_2_O (5 mmol) were dissolved into 75 mL deionized water, then mixed with 75 mL of catechol (20 mM) sufficiently. When catechol was mixed with iron solution (pH 2.87), the polymerization of catechol catalyzed by Fe^3+^ happened, forming polycatechol, which was black coarse precipitates [30]. Simultaneously, Fe^3+^ ions were chemically adsorbed on polycatechol through complexation and served as nucleation precursors. The mixture was standing for 30 min and then was added into 100 mL of ammonium hydroxide (3.3 M) rapidly, aging for 120 min under vigorous stirring. The magnetic nanoparticles in situ grew to form aggregations on the surface of polycatechol chains. Couples of Fe_3_O_4_/PCC chains combined with each other tightly to form Fe_3_O_4_/PCC MNPs. The whole synthesis processes were performed at ambient atmosphere, without any protective atmosphere. The black magnetic nanoparticles were separated by an external magnet and washed with deionized water until neutral pH and dried in a vacuum oven at 50 °C for 24 h. Fe_3_O_4_ MNPs were synthesized with the same procedures as mentioned above without adding catechol. All the products were stored in a desiccator under ambient temperature for further experiments.

Magnetic properties were measured at room temperature on a magnetic property measurement system (MPMS XL-7, Quantum Design, America). Thermogravimetric analysis (TGA) was carried out for powder samples using a TGA/DSC 1 thermogravimetric analyzer (TGA) (Mettler-Toledo, Switzerland) under N_2_ environment at a heating rate of 5 °C min^− 1^. The zeta potentials of catalyst suspensions at different pH were determined by a Malvern 3000 Zetasizer.

### Batch adsorption experiments

Sorption isotherm experiments were carried out by shaking 25 mg Fe_3_O_4_/PCC MNPs in 25 mL solution with varied adsorbates, with initial adsorbate concentration varying from 0.02 mM to 0.4 mM. The mixture was continuously shaken on a shaker at 180 rpm under controlled temperature of 30 °C until reaching equilibrium. The solution pH was adjusted by using 1.0 M H_2_SO_4_ or 1.0 M NaOH solutions. After adsorption, the adsorbent was separated from the solution under magnetism, and then the supernatant liquid was measured at the maximum absorbance of each dye by a UV-vis spectrophotometer.

Furthermore, the adsorption kinetics of the processes were studied. 100 mg Fe_3_O_4_/PCC MNPs were suspended into 100 mL 0.1 mM solutions of adsorbates, and then shaken at 180 rpm under pH 6.0 and 30 °C. At different time intervals, 0.5 mL of suspension sample was withdrawn and immediately separated by an external magnetism and the supernatant liquid was collected for analysis.

The influences of pH value and temperature on adsorption of cationic dyes were also studied. The typical batch adsorption experiment was carried out as follows: 50.0 mg of Fe_3_O_4_/PCC MNPs was dispersed in 50.0 mL of cationic dyes solution and then was shaken on a shaker with a speed of 180 rpm.

All the adsorption experiments were carried out in duplicate. The adsorption capacity of each dye in the adsorption system, q_*e*_, was calculated according to Eq. (1):

1 $$ {q}_e=\left({C}_i-{C}_e\right)\ V/{M}_s $$

Where *q*_*e*_ (mg g^− 1^) is the adsorption capacity, *C*_*e*_ (mM) is the equilibrium concentration in the aqueous phase, *Ci* (mM) is the initial aqueous phase concentration, *V* (L) is the volume of solution and *M*_*s*_ (g) is the mass of solid adsorbent.

## Results and discussion

### Characterization of Fe_3_O_4_/PCC MNPs

**Fig. 2:**
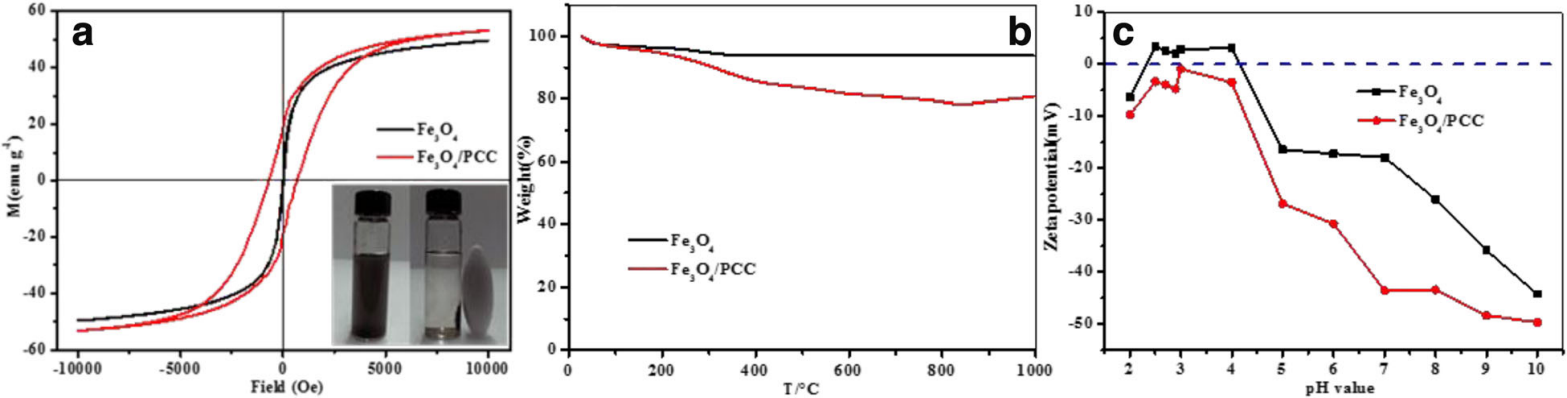
**a** Magnetization behavior of Fe_3_O_4_ MNPs and Fe_3_O_4_/PCC MNPs at room temperature. **b** Thermogravimetry (TGA) curves of Fe_3_O_4_ MNPs and Fe_3_O_4_/PCC MNPs. **c** zeta potentials of Fe_3_O_4_/PCC magnetic nanoparticles. In Fig. 2, the blank lines represent the nature of Fe_3_O_4_ MNPs, and the red lines are on behalf of the property of Fe_3_O_4_/PCC MNPs

Figure 2a shows the magnetic hysteresis loops determined at room temperature for Fe_3_O_4_ and Fe_3_O_4_/PCC MNPs. The saturation magnetization values of Fe_3_O_4_/PCC MNPs were 53.5 emu g^− 1^, higher than that of Fe_3_O_4_ (49.6 emu g^− 1^), suggesting that they could be easily separated by an external magnetic field [33]. The particle size, spin canting phenomenon, size effect, and others, are related to the saturation magnetization of the ferrite nanoparticles [34]. The modification of polycatechol makes the Fe_3_O_4_/PCC MNPs much higher in crystallization, and smaller in particle size than Fe_3_O_4_ MNPs, which could be result in the higher saturation magnetization of Fe_3_O_4_/PCC MNPs than Fe_3_O_4_ MNPs. Furthermore, higher saturation magnetization of the prepared Fe_3_O_4_/PCC MNPs could also be attributed to the surface effect, sometimes called the “dead surface”. The dead surface is associated with disorder of surface spin [35].

The thermal behaviors of Fe_3_O_4_ and Fe_3_O_4_/PCC MNPs were further investigated by thermogravimetric analysis (TGA) (Fig. 2b). For the TGA curve of Fe_3_O_4_ MNPs, the weight loss (6.5%) below 150 °C was due to the loss of physically adsorbed water. For the curve of Fe_3_O_4_/PCC MNPs, the weight loss (5.2%) below 150 °C was due to the loss of physically adsorbed water, the weight loss (9.4%) from 150 °C to 400 °C was ascribed to the loss of oxygen-containing functional groups, the weight loss (6.8%) from 400 °C to 800 °C was mainly attributed to the burning of carbon, and a slight weight gain (2.3%) over 800 °C was due to the oxidization of Fe_3_O_4_ to γ-Fe_2_O_3_ [36]. The Fe_3_O_4_/PCC MNPs exhibited a lower thermal stability rather than Fe_3_O_4_, due to the modification of polycatechol [37].

Figure 2c shows the zeta potentials of the Fe_3_O_4_ and Fe_3_O_4_/PCC suspensions at various pH values. As shown in Fig. 2c, the isoelectric point of Fe_3_O_4_ was 4.2, while the surface of Fe_3_O_4_/PCC MNPs possessed negative charges in the pH range of 3.0–10.0, which could be due to the electronegativity of phenolic hydroxyl group in polycatechol. Moreover, the surface charge density of Fe_3_O_4_/PCC MNPs increased as the pH increased from 3.0 to 10.0. The negative charges of Fe_3_O_4_/PCC MNPs also prevented nanoparticles from agglomeration.

### Selective adsorption of Fe_3_O_4_/PCC MNPs

**Fig. 3:**
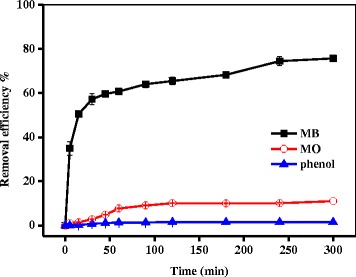
The removal efficiency of MB, MO and phone adsorbed by Fe_3_O_4_/PCC MNPs. As shown as Fig. 3, the blank line represent the removal efficiency of MB, the red line reperesent the removal of MO, and the blue line represent the removal of phonel

The adsorption properties of the Fe_3_O_4_/PCC MNPs towards cationic dyes, anionic dyes and phenol from aqueous solution were investigated in detail. Figure 3 shows the removal efficiencies of MB as a kind of cationic dye, MO as a kind of anionic dye and phenol adsorbed onto Fe_3_O_4_/PCC MNPs. It was observed that the removal efficiency of MB was 75.7%. However, the removal efficiencies of MO was 10.9% only, and the removal efficiency of phenol was 1.5% only. The results indicated that the Fe_3_O_4_/PCC MNPs selectively adsorbed cationic dye, due to the electrostatic interaction (Fig. 2c).

### Adsorption kinetics of cationic dyes

Adsorption kinetics of five cationic dyes on Fe_3_O_4_/PCC MNPs were studied using two kinetic models, namely the pseudo-first-order model and the pseudo-second-order model (Fig. 3). In the pseudo-first-order kinetic model, the rate constant of dyes adsorption is expressed as [38]:

2 $$ Ln\left({q}_e-{q}_t\right)=\mathit{\ln}\left({q}_e\right)\hbox{--} {k}_1\ t $$

where *q*_*e*_ and *q*_*t*_ are the amounts of dyes adsorbed (mg g^− 1^) at equilibrium and at any instant of time *t* (min), respectively, and *k*_*1*_ is the rate constant of pseudo-first-order adsorption (min^− 1^).

The pseudo-second-order kinetic model is described by the formula [39]:

3 $$ t/{q}_t= 1/{k}_{ad}\ {q_e}^2+ 1/{q}_e $$

**Fig. 4:**
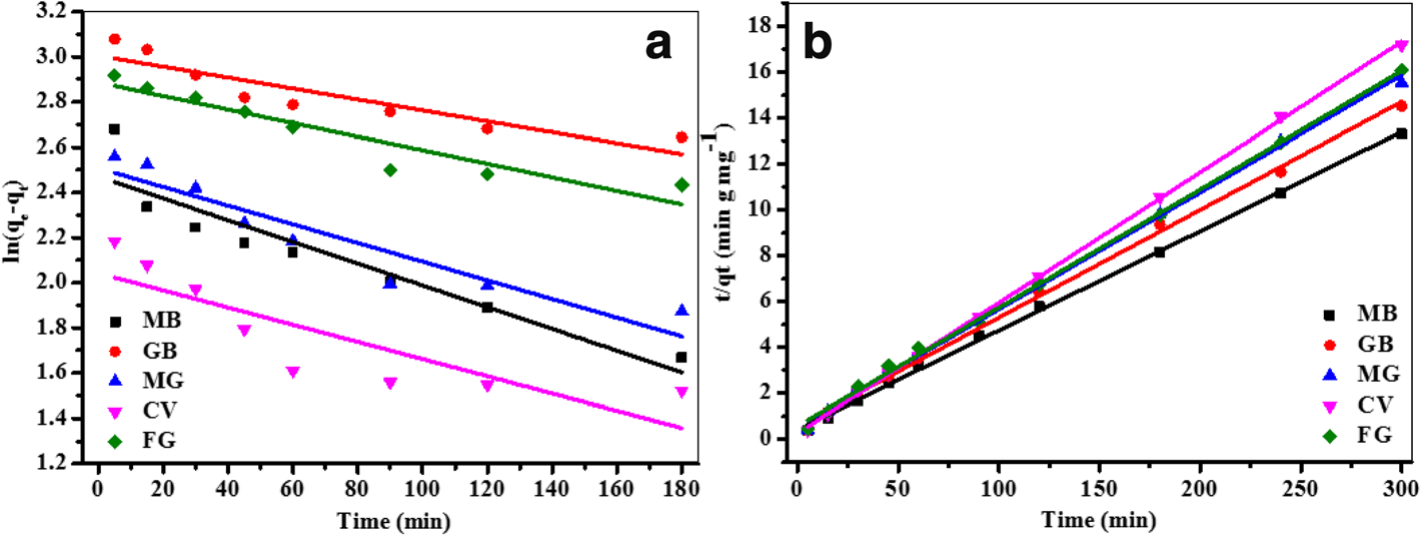
Adsorption of cationic dyes on Fe_3_O_4_/PCC MNPs (**a**) pseudo-second-order kinetics (**b**) pseudo-first-order kinetics. As shown as Fig. 4, the blank line represent the adsorption of MB, the red line reperesent the adsorption of GB, the blue line represent the adsorption of MG, the magenta represent of CV adsorption, and the olive is on behalf of the adsorption of FG

Where *q*_*e*_ and *q*_*t*_ are, respectively, the amount of dyes adsorbed at the equilibrium and time *t* (mg g^− 1^); and *k*_*ad*_ is the pseudo-second-order rate constant for the adsorption process (mg g^− 1^ min^− 1^). The parameter values for each model were calculated from the linear least square method and the correlation coefficients were presented in Table 1. The results showed that all the adsorption kinetics of these five cationic dyes on Fe_3_O_4_/PCC MNPs could be well described by pseudo-second-order kinetics model with high correlation coefficient (R^2^ > 0.997) and the rate constants (*k*_*ad*_) were calculated to 0.043, 0.047, 0.051, 0.057, 0.052 g mg^− 1^ mL^− 1^, corresponding to MB, GB, MG, CV and FG, respectively (Fig. 4 and Table 1). Moreover, the adsorption capacity of MB on Fe_3_O_4_/PCC MNPs was significantly improved, comparing to that of Fe_3_O_4_ MNPs (Additional file 1: Figure S1). The main reason was the electrostatic attractions between the positive charge of cationic dyes and the negative charge of Fe_3_O_4_/PCC MNPs.

**Table 1:** Kinetics parameters for the adsorption of cationic dyes on Fe_3_O_4_/PCC MNPs

Cationic dyes	pseudo-first-order kinetics			pseudo-second-order kinetics		
	*q*_*e*_	*k*	*R* ^*2*^	*q*_*e*_	*k*	*R* ^*2*^
MB	11.83	0.0048	0.859	23.12	0.043	0.999
GB	20.17	0.0024	0.798	21.26	0.047	0.997
MG	12.28	0.0041	0.868	19.61	0.051	0.998
CV	7.71	0.0038	0.682	17.57	0.057	0.999
FG	17.92	0.0030	0.873	19.38	0.052	0.998

### Adsorption isotherms of different cationic dyes

The adsorption isotherm played a significant role in evaluating the adsorption properties of Fe_3_O_4_/PCC MNPs [40]. To depict the adsorption process thoroughly, two well-known isotherm equations, Langmuir and Freundlich equations (Eqs. (4) and (5)), were applied [41].

Langmuir equation:

4 $$ {C}_e/{q}_e={C}_e/{q}_m+ 1/{K}_L\ {q}_m $$

where *q*_*e*_ (mg g^− 1^) is the equilibrium adsorption capacity of dye on the adsorbent; *C*_*e*_ (mg L^− 1^) is the equilibrium dye concentration in solution; *q*_*m*_ (mg g^− 1^), the maximum capacity of the adsorbent; and *K*_*L*_ (L mg^− 1^), the Langmuir constant.

Freundlich equation:

5 $$ {q}_e={K}_F\ {C_e}^{1/n} $$

Where *q*_*e*_ and *C*_*e*_ are defined to be the same as above; *K*_*F*_ (L mg^− 1^) is the Freundlich constant; and n is the heterogeneity factor.

**Fig. 5:**
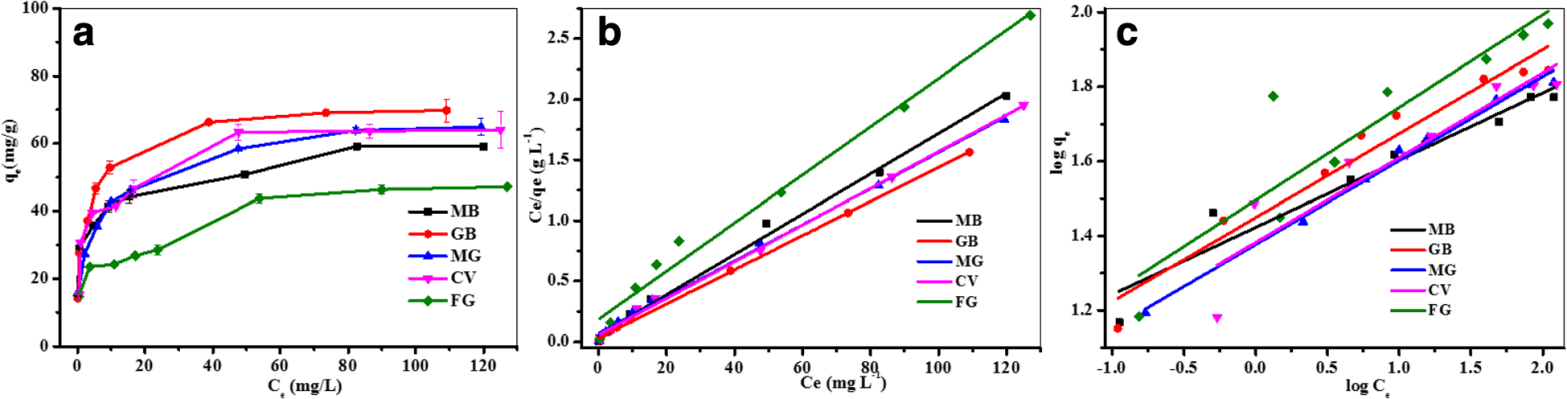
**a** Isotherms of cationic dyes adsorption on Fe_3_O_4_/PCC MNPs (**b**) Langmuir and (**c**) Freundlich adsorption isotherm models for organic dyes by Fe_3_O_4_/PCC MNPs. As shown as Fig. 5, the blank line represent the adsorption of MB, the red line reperesent the adsorption of GB, the blue line represent the adsorption of MG, the magenta line represent of CV adsorption, and the olive line is on behalf of the adsorption of FG

Figure 5 shows the adsorption isotherms of cationic dyes on Fe_3_O_4_/PCC MNPs. The results indicated that the adsorption of the five cationic dyes all fitted better with Langmuir equation than with Freundlich equation according to the correlation coefficients. The maximum adsorption capacities (*q*_*m*_) for these dyes were worked out by the Langmuir equation which were listed in Table 2. The *q*_*m*_ for cationic dyes: MB, GB, MG, CV and FG were 60.06, 70.97, 66.84, 66.01 and 50.27 mg g^− 1^, respectively. The fitted Langmuir model assumed that the single pollutant bonded to a single site on the adsorbent and that all surface sites on the adsorbents had the same affinity for pollutant and no interactions between pollutant [42].

**Table 2:** Parameters of adsorption isotherm models parameters for the adsorption of dyes on Fe_3_O_4_/PCC MNPs

Cationic dyes	Langmuir model			Freundlich model		
	q_e_	b	R^2^	q_e_	n	R^2^
MB	60.06	0.31	0.995	26.48	5.54	0.928
GB	70.97	0.47	0.999	28.12	4.43	0.943
MG	66.84	0.22	0.997	23.81	4.44	0.990
CV	66.01	0.28	0.997	24.14	4.39	0.852
FG	50.27	0.34	0.983	31.33	4.02	0.796

### Effect of temperature on cationic dyes adsorption

**Fig. 6:**
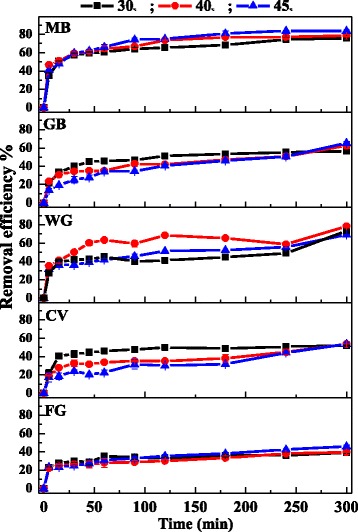
Effect of temperature on the adsorption of cationic dyes on Fe_3_O_4_/PCC MNPs. As shown as Fig. 6, the blank line represent that the temperature is 30 °C, the red line reperesent that the temperature is 40 °C, and the blue line represent the temperature is 45 °C

The effect of temperature on the adsorption of cationic dyes was shown in Fig. 6. As can be seen, the removal efficiency of MB increased with rising temperature (30–45 °C), and it reached up to 84% at 45 °C, which suggested that the adsorption of MB on Fe_3_O_4_/PCC was an endothermic process. While the removal efficiency of GB and CV decreased with rising temperature, suggesting an exothermic reaction for the adsorption of GB and CV, which indicated the sorption processes were mainly physical adsorption. Furthermore, the reaction temperature had little effect on the adsorption of WG and FG. The effect of reaction temperature on the adsorption of five cationic dyes was different, mainly because of the different structure of dyes and the hole of the MNPs. When the holes of the MNPs are too small to be get into, the adsorbate molecules have to go over the high barrier to get into the hole. Since the holes are small and the diffusion is blocked, the adsorption process is more unstable, resulting in higher energy and the process is endothermic. Otherwise the process is exothermic.

### Effect of pH on cationic dyes adsorption

**Fig. 7:**
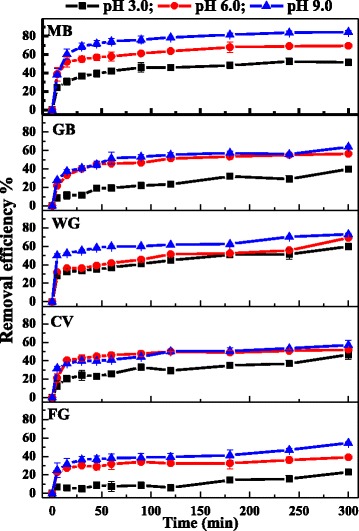
Effect of initial pH on the removal of cationic dyes on Fe_3_O_4_/PCC MNPs the temperature is 30 °C. As shown as Fig. 7, the blank line represent that the solution pH is 3.0, the red line reperesent that the solution pH is 6.0, and the blue line represent that the solution pH is 9.0

The pH of the aqueous solution was an important factor that affects the dye-adsorption process, because it influenced the surface charge of an adsorbent and the ionization behavior of both the adsorbent and dye [43]. The effect of pH on the removal of cationic dyes was studied at a dye concentration of 0.1 mM at 30 °C and at pH values from 3.0 to 9.0. As shown in Fig. 7, the removal efficiency of cationic dyes increased with increasing pH value. Because the Fe_3_O_4_/PCC MNPs possessed negative charge, and their surface charge density increased with higher pH (Fig. 2c), cationic dyes were adsorbed on Fe_3_O_4_/PCC MNPs through the electrostatic attractions between the positive charge of cationic dyes molecules and the negative charge of Fe_3_O_4_/PCC MNPs. As the pH increases, the electrostatic attraction between the negatively charged surface of the Fe_3_O_4_/PCC composite and cationic dyes molecule increased, resulting in the increase in the adsorption capacity of cationic dyes. Therefore, the elevated pH helped the removal of cationic dyes by Fe_3_O_4_/PCC MNPs.

### Effect of coexisting cations on MB adsorption

**Fig. 8:**
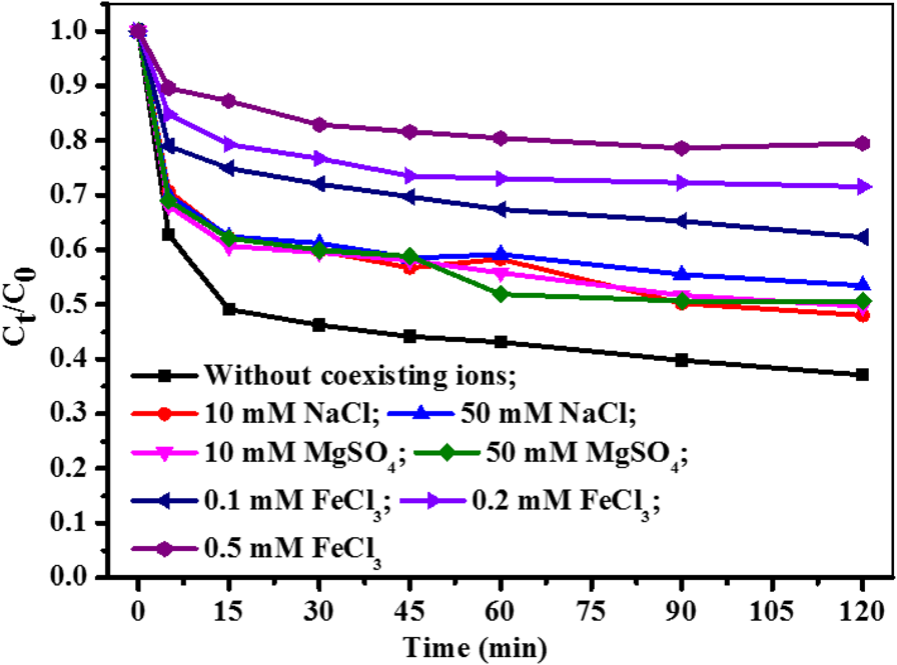
Effect of coexisting cations and ionic strength on the adsorption of MB on Fe_3_O_4_/PCC MNPs. As shown as Fig. 8, the blank line represent the adsorption of MB without any coexisting ions, the red line reperesent the effect on MB adsorption with 10 mM NaCl, the blue line reperesent the effect on MB adsorption with 50 mM NaCl, the magenta line reperesent the effect on MB adsorption with 10 mM MgSO_4_, the olive line reperesent the effect on MB adsorption with 50 mM MgSO_4_, the navy line reperesent the effect on MB adsorption with 0.1 mM FeCl_3_, the violet line reperesent the effect on MB adsorption with 0.2 mM FeCl_3_, the purple line reperesent the effect on MB adsorption with 0.1 mM FeCl_3_

Dye effluents always contained a large variety of coexisting ions, which might affect the dye adsorption process [4]. In this study, three commonly coexisting salts, NaCl, MgSO_4_ and FeCl_3_ were selected to study the effect of coexisting cations and their ionic strength on MB adsorption onto Fe_3_O_4_/PCC MNPs with the results presented in Fig. 8. As can be seen, Na^+^, Mg^2+^ and Fe^3+^ all suppressed MB adsorption due to the competition adsorption between cations and MB on the adsorptive sites of Fe_3_O_4_/PCC MNPs. Moreover, the removal efficiency of MB decreased from 63% to 20% with Fe^3+^ concentration increasing from 0.1 mM to 0.5 mM. Such competitive adsorption was widely reported in the literature [44]. The results further confirmed the electrostatic adsorption of MB on Fe_3_O_4_/PCC MNPs.

### Recycle of the adsorbent

**Fig. 9:**
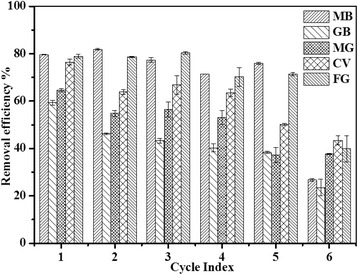
The histogram of removal efficiency of cationic dyes by Fe_3_O_4_/PCC MNPs adsorption ([Fe_3_O_4_/PCC] = 1.0 g L^− 1^, [dyes]_0_ = 0.1 mM, pH 6.0, t = 300 min)

After adsorption, Fe_3_O_4_/PCC MNPs could be regenerated with ethanol desorption at pH 4.0 for 12 h and washed with deionized water to neutral condition. The Fe_3_O_4_/PCC MNPs could be regenerated and reused for five times. Figure 9 shows the adsorption performance of the regenerated Fe_3_O_4_/PCC MNPs for cationic dyes. The removal efficiency of cationic dyes decreased gradually during the first adsorption-desorption cycle to the fifth cycle. At the sixth cycle, the removal efficiency of MB, GB, MG, CV and FG decreased dramatically to 27%, 23%, 37%, 43% and 39%, respectively. Notably, the presence of magnetic nanoparticles facilitated separation and recovery of the adsorbent. It indicates that the Fe_3_O_4_/PCC MNPs has a certain economic feasibility.

## Conclusion

In conclusion, a new magnetic nano-adsorbent (Fe_3_O_4_/PCC MNPs) was successfully prepared with active adsorption sites for removing cationic dyes from aqueous solution. The introduction of polycatechol in the structure of Fe_3_O_4_/PCC MNPs performed amazing advantages, including preventing nanoparticles from agglomeration and improving adsorption behavior of the MNPs. The electrostatic interaction was found to be the main force of the adsorption behavior for the cationic dyes. The adsorption process was well-described by pseudo-second-order kinetics and Langmuir isotherm models, respectively. The results demonstrated that Fe_3_O_4_/PCC MNPs showed potential application for cationic dyes removal in industrial effluents.

## Additional file

Additional file 1: **Figure S1**. Adsorption removal efficiency of MB on Fe3O4 and Fe3O4/PCC MNPs, inset is the adsorption capacity of MB ([Fe3O4/PCC] = 1.0 g L-1, [Fe3O4] = 1.0 g L-1, [MB] = 0.1 mM, pH = 6.0, T = 30 °C). (DOC 45 kb)

## Abbreviations

CR: Congo red
CTAB: Cetyltrimethylammonium bromide
Fe_3_O_4_/PCC: Fe_3_O_4_/polycatechol
Gly: Glycine
GPTMS: 3-glycidoxypropyltrimethoxysilane
MB: Methylene blue
MNPs: Magnetic nanoparticles
PCC: Polycatechol
TGA: Thermogravimetric analysis
