# Supplementary material for: Facile synthesis of surface-functionalized magnetic nanocomposites for effectively selective adsorption of cationic dyes
Source: Nanoscale Res Lett. 2018 Apr 12;13:99. doi: 10.1186/s11671-018-2476-7 (PMC5897273; doi:10.1186/s11671-018-2476-7)
Supplement: Supplementary file 1 — Figure S1. Adsorption removal efficiency of MB on Fe3O4 and Fe3O4/PCC MNPs, inset is the adsorption capacity of MB ([Fe3O4/PCC] = 1.0 g L-1, [Fe3O4] = 1.0 g L-1, [MB] = 0.1 mM, pH = 6.0, T = 30 °C). (DOC 45 kb) [file 11671_2018_2476_MOESM1_ESM.doc]

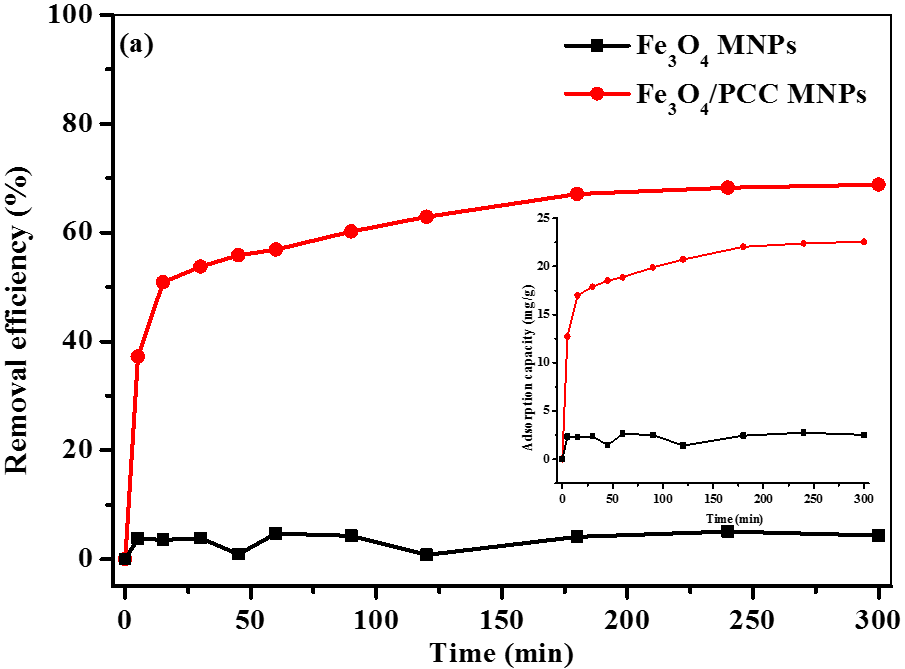


Fig. S1 Adsorption removal efficiency of MB on Fe_3_O_4_ and Fe_3_O_4_/PCC MNPs, inset is the adsorption capacity of MB ([Fe_3_O_4_/PCC] = 1.0 g L^-1^, [Fe_3_O_4_] = 1.0 g L^-1^, [MB] = 0.1 mM, pH = 6.0, T = 30 °C)
